# IL-10 Overexpression After BCG Vaccination Does Not Impair Control of *Mycobacterium tuberculosis* Infection

**DOI:** 10.3389/fimmu.2022.946181

**Published:** 2022-07-22

**Authors:** Catarina M. Ferreira, Consuelo Micheli, Palmira Barreira-Silva, Ana Margarida Barbosa, Mariana Resende, Manuel Vilanova, Ricardo Silvestre, Cristina Cunha, Agostinho Carvalho, Fernando Rodrigues, Margarida Correia-Neves, António Gil Castro, Egídio Torrado

**Affiliations:** ^1^ Life and Health Sciences Research Institute (ICVS), School of Medicine, University of Minho, Braga, Portugal; ^2^ ICVS/3B’s - PT Government Associate Laboratory, Braga/Guimarães, Portugal; ^3^ Instituto de Investigação e Inovação em Saúde (i3S), Universidade do Porto, Porto, Portugal; ^4^ Instituto de Biologia Molecular e Celular (IBMC), Universidade do Porto, Porto, Portugal; ^5^ Instituto de Ciências Biomédicas de Abel Salazar, Universidade do Porto (ICBAS-UP), Porto, Portugal; ^6^ Division of Infectious Diseases, Department of Medicine Solna, Karolinska Institutet, Stockholm, Sweden

**Keywords:** tuberculosis, vaccination, IL-10, granuloma, tertiary lymphoid follicles

## Abstract

Control of tuberculosis depends on the rapid expression of protective CD4^+^ T-cell responses in the *Mycobacterium tuberculosis* (Mtb)-infected lungs. We have recently shown that the immunomodulatory cytokine IL-10 acts intrinsically in CD4^+^ T cells and impairs their parenchymal migratory capacity, thereby preventing control of Mtb infection. Herein, we show that IL-10 overexpression does not impact the protection conferred by the established memory CD4^+^ T-cell response, as BCG-vaccinated mice overexpressing IL-10 only during Mtb infection display an accelerated, BCG-induced, Ag85b-specific CD4^+^ T-cell response and control Mtb infection. However, IL-10 inhibits the migration of recently activated ESAT-6-specific CD4^+^ T cells into the lung parenchyma and impairs the development of ectopic lymphoid structures associated with reduced expression of the chemokine receptors CXCR5 and CCR7. Together, our data support a role for BCG vaccination in preventing the immunosuppressive effects of IL-10 in the fast progression of Mtb infection and may provide valuable insights on the mechanisms contributing to the variable efficacy of BCG vaccination.

## Introduction

Tuberculosis (TB), caused by *Mycobacterium tuberculosis* (Mtb), is a leading cause of infectious deaths worldwide second only to COVID-19 (1). In this regard, the COVID-19 pandemic reversed years of progress in reducing the number of people who die from TB globally. Indeed, in the last years there has been a significant decline in the notification of TB cases and interventions, which has caused, for the first time in more than a decade, an increase in the number of TB deaths (1). These epidemiological changes are furthermore expected to have serious consequences for the global control of TB in the coming years, with over 1.4 million TB deaths and 6.3 million cases of active TB estimated to occur by 2025 (1).

Vaccination is the most sustainable and cost-effective answer to this threat. However, *Mycobacterium bovis* Bacille Calmette-Guérin (BCG), currently the only licensed TB vaccine, has been shown to be effective in preventing disseminated forms of pediatric TB but not adult pulmonary disease (2–4). This limited protection against pulmonary disease is, in part, a consequence of the short-lived immunity conferred by BCG, which typically wanes by adolescence (5). However, there is now revived optimism in BCG revaccination to control pulmonary TB in adolescents and adults, with a recent study showing an efficacy of BCG revaccination of 45.4% against Mtb infection (6). Despite these promising results, vaccines with efficacy of 50% or more are considered necessary to reach the target set by the WHO End TB strategy (7). It is therefore critical to understand why BCG is ineffective against pulmonary disease in order to devise novel strategies to improve its efficacy and the efficacy of novel vaccines.

Interleukin-10 (IL-10) has been shown to increase the susceptibility to TB in both humans and animal models, an event highly associated with impaired CD4^+^ T-cell responses (8–13). Using a mouse model of controlled IL-10 overexpression, we recently demonstrated that IL-10 overexpression before the onset of the acquired immune response to Mtb promotes the differentiation of vasculature-associated CD4^+^ T cells with reduced ability to migrate to the lung parenchyma and engage with infected phagocytes (14). By showing that IL-10 overexpression during the chronic stages of the infection does not impact control of infection, our data demonstrate that the reduced migration of CD4^+^ T cells to the lung parenchyma is the critical mechanism whereby IL-10 antagonizes control of Mtb infection (14). Indeed, the parenchyma location of CD4^+^ T cells is crucial to controlling Mtb infection, as control of bacterial growth occurs only after T cells engage directly with infected phagocytes (15, 16). Furthermore, CD4^+^ T cells in the lung parenchyma promote the development of a more protective granuloma characterized by the presence of organized B-cell aggregates, reminiscent of ectopic lymphoid structures (17). Indeed, the formation of these structures has been associated with control of infection in animal models (18–20) and with controlled latent TB in humans (17, 21). In this regard, while B-cell follicles themselves do not appear to be protective in the mouse model of TB, the cytokines and chemokines associated with the development of these cellular aggregates are critical for T-cell location within the lungs, enabling them to engage with infected phagocytes and induce Mtb control (17, 22, 23). Accordingly, the expression of CXCL13, CCL19, and CCL21 as well as their receptors, CCR7 and CXCR5, has been shown to be crucial for the formation of ectopic lymphoid structures and control of Mtb infection (17, 23, 24).

We recently demonstrated that IL-10 antagonizes the control of Mtb infection by driving the differentiation of CD4^+^ T cells with reduced ability to migrate to the lung parenchyma (14). Therefore, in this work we investigated if the impact of IL-10 in the CD4^+^ T-cell response and control of Mtb infection is similar in vaccinated mice as we previously reported in naive mice (14). To this end, we used our mouse model of controlled IL-10 overexpression (pMT-10) (14, 25) which were vaccinated with BCG 2 months before being infected with Mtb through the aerosol route. We then induced IL-10 overexpression and determined the ability of pMT-10 mice to mount a protective response and control Mtb infection. Our data show that BCG vaccination prevents the early disease progression induced by IL-10 overexpression in unvaccinated mice, associated with an early accumulation of BCG-induced Ag85b-specific CD4^+^ T cells in the lung. Despite this, BCG vaccination does not overcome the deficient migration of Mtb-specific (ESAT-6) CD4^+^ T cells into the lung parenchyma, as previously described in naïve mice (14). Importantly, despite the improved control of infection there is a reduced formation of B-cell aggregates in the lungs of BCG-vaccinated pMT-10 mice. These data show that IL-10 overexpression has a differential impact in the primary and secondary responses. These findings may be relevant to unravel new mechanisms contributing to the variable efficacy of BCG vaccination in preventing pulmonary TB, particularly in individuals with intrinsically higher levels of IL-10.

## Materials and Methods

### Mice

C57BL/6 (B6) mice, originally purchased from Charles River Laboratory (Barcelona, Spain), were bred and maintained at the ICVS animal facility. pMT-10 mice in a B6 background were generated by Drs. António G. Castro and Paulo Vieira, as previously described (25). Briefly, mouse IL-10 cDNA cloned in the p169ZT vector, carrying the sheep metalloprotein 1a promotor, a β-globin splice site, and the SV40 polyadenylation signal, was injected in B6 eggs. The identification of transgenic founders was determined by PCR using sheep metalloprotein and IL-10-specific primers.

To induce IL-10 overexpression, the drinking water of pMT-10 mice was supplemented with a 2% sucrose solution containing 50 mM of zinc sulfate (25). This supplementation induces a rapid increase in the circulating levels of IL-10, which are maintained until zinc sulfate withdraws (25). B6 mice were maintained in the same condition as pMT-10 mice, including drinking water supplemented with 2% sucrose solution containing 50 mM of zinc sulfate.

Both male and female mice between 6 and 12 weeks of age were used for experimental procedures. Mice euthanasia was carried out by controlled CO_2_ inhalation.

### BCG Vaccination, Mtb Aerosol Infection, and Bacterial Burden Determination

Mice were subcutaneously vaccinated with 1 × 10^6^ CFU of *M. bovis* BCG Pasteur and rested for 60 days prior to Mtb aerosol infection.

The H37Rv strain of Mtb used in this study was originally obtained from the Trudeau Institute (Saranac Lake, NY). Mtb H37Rv was first grown to log phase in Middlebrook 7H9 broth supplemented with 10% oleic acid/albumin/dextrose/catalase (OADC), 0.2% glycerol, and 0.05% Tween 80 and then subcultured in Proskauer-Beck medium with 0.05% Tween-80 to mid-log phase before being frozen at -80°C. These frozen stocks were quantified and used to infect mice with a low dose of bacteria (~75 CFUs) using the Glas-Col airborne infection system, as previously described (14).

At different time-points after infection, the lungs were aseptically excised and used to prepare lung single-cell suspensions for flow cytometry analysis or homogenized for CFU counts. Organ homogenates were serially diluted and plated onto Middlebrook 7H11 agar (BD Biosciences, San Jose, CA, USA) for 3 weeks at 37°C, at which point CFUs were counted.

### Lung Single-Cell Suspensions

Aseptically excised lungs were sectioned and incubated at 37°C for 30 min with collagenase D (0.7 mg/ml, Sigma, St. Louis, MO, USA). Samples were then disrupted into a single-cell suspension by passage through a 70-μm nylon cell strainer (BD Biosciences). Lung single-cell suspensions were then treated with erythrocyte lysis buffer (0.87% of NH_4_Cl) and processed over 40:80% Percoll (GE Healthcare, Chicago, IL, USA). The resulting cell suspension was washed twice and counted.

For intravital flow cytometry analysis, mice received intravenously APC-labeled anti-CD45 antibody 3 min before euthanasia (26). Lung single-cell suspensions prepared as described above were stained with fluorochrome-conjugated antibodies for 30 min on ice. For intracellular cytokine detection, cells were cultured in 5 µg/ml of Ag85b_240-254_ or ESAT-6_1-20_ peptide for 1.5 h before 10 µg/ml Brefeldin A (Sigma-Aldrich) was added to the culture for an additional 3.5 h. Cells were analyzed by flow cytometry using the following antibodies: CD3-Brilliant Violet 605 (145-2C11, BioLegend, San Diego, CA, USA), CD4-APC-Cy7 (GK1.5, BioLegend), CD44-PercPCy5.5 (IM7, BioLegend), CD45-Brilliant Violet 510 (30-F11, BioLegend), CD45.2-APC (104, BioLegend), and IFNγ-PE-Cy7 (XMG1.2, BioLegend). Data were acquired on a LSRII flow cytometer (BD Biosciences) with Diva Software and analyzed using FlowJo software (BD Biosciences). The total number of cells for each population was calculated by taking into account the percentage of cells determined by flow cytometry and the total number of cells per lung.

### Histology and Immunohistochemistry

The right upper lobe of each lung was inflated with 4% paraformaldehyde (PFA) and processed routinely for light microscopy with hematoxylin and eosin stain. Morphometric analysis was performed in a blinded manner using ImageJ software (version 1.50e; NIH, Bethesda, MD, USA). The percentage of the lung inflamed for each sample was calculated by dividing the cumulative area of inflammation by the total lung surface area. The percentage of B-cell aggregates in the granuloma was calculated by dividing the area of lung tissue stained positive for B220 by the area of inflammation in the lung.

Immunofluorescence was performed on formalin-fixed lung sections, as described previously (14). Sections were stained with either purified rabbit polyclonal anti-CD3e (ab185811; Abcam, Cambridge, MA, USA) or CD45R (RA3-6B2; Abcam) followed by streptavidin–Alexa Fluor 488 (Invitrogen). SlowFade Gold antifade with DAPI (Invitrogen) was used to counterstain tissues and to reveal nuclei. Representative images were obtained with an Olympus BX61 microscope and were recorded with the DP70 digital camera using the cell^P software.

### Real-Time PCR Analysis

Total RNA from infected lungs was extracted using TRIzol reagent (Invitrogen) following the manufacturer’s instructions. cDNA was generated from 1 μg of total RNA using the GRS cDNA Synthesis Master Mix (GRiSP, Porto, Portugal) following the manufacturer’s instructions. The resultant cDNA template was used to quantify the expression of target genes by real-time (RT)-PCR (Bio‐Rad CFX96 Real‐Time System with C1000 Thermal Cycler) using the following protocol: one cycle of 95°C for 3 min, followed by 40 cycles of a two-stage temperature profile of 95°C for 3 s and 60°C for 30 s. Gene expression was normalized to ubiquitin mRNA levels using the ΔCt method. Target gene mRNA expression was quantified using SYBR Green (Thermo Scientific, Waltham, MA, USA) and specific oligonucleotides (**Table 1**; Invitrogen, Carlsbad, CA).

**Table 1:** Primers used to determine gene expression by RT-PCR.

Target gene	Forward sequence	Reverse sequence
*Ubq*	TGGCTATTAATTATTCGGTCTG	GCAAGRGGCTAGAGTGCAGAGTA
*Ccl19*	CCTGCTGTTGTGTTCACCACA	TGTTGCCTTTGTTCTTGGCA
*Ccl21*	TCCAACTCACAGGCAAAGAGG	GGCCGTGCAGATCTAATGGTT
*Cxcl13*	CTCCAGGCCACGGTATTCTG	CCAGGGGGCGTAACTTGAAT
*Ccr7*	GTACGAGTCGGTGTGCTTC	GGTAGGTATCCGTCATGGTCTTG
*Cxcr5*	CAGACTTCATGCCAGACCTTCA	CCAATGCTGGTTAGGTTGGAAC

### Statistical Analysis

Differences between groups were determined using one-way ANOVA. Differences were considered significant for p ≤ 0.05.

## Results

### BCG Vaccination Prevents the Fast Progression of Mtb Infection Induced by IL-10

Our previous data demonstrated that, in an IL-10-enriched environment CD4^+^ T cells differentiate into a vasculature-associated phenotype with reduced ability to migrate into the parenchyma and infiltrate the Mtb lung lesion (14). This migratory deficit prevented the control of Mtb, resulting in exacerbated bacterial proliferation and lung pathology (14). As early protection conferred by BCG vaccination has been shown to depend on lung-resident memory CD4^+^ T cells (16, 27), we used our pMT-10 mouse model to test the impact of IL-10 in vaccinated hosts. To do this, we vaccinated B6 and pMT-10 mice with BCG through the subcutaneous route. Two months after vaccination, mice were infected with Mtb through the aerosol route, and the expression of IL-10 in pMT-10 mice initiated by supplementing the drinking water with 50 mM of zinc sulfate, as previously described (14, 25). Vaccinated and unvaccinated B6 mice maintained in the same conditions as pMT-10 mice were used as controls.

**Figure 1:**
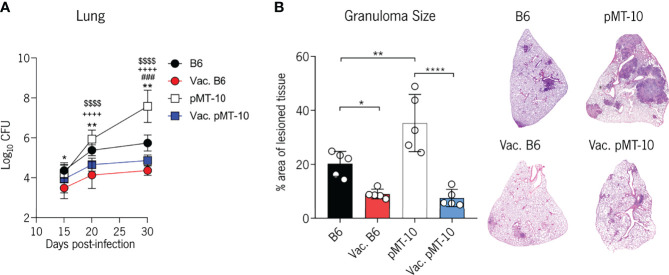
BCG vaccination prevents the extensive Mtb proliferation and lung pathology induced by IL-10 overexpression. B6 and pMT-10 mice were subcutaneously vaccinated with 1 × 10^6^ BCG. Unvaccinated mice were used as controls. Two months after vaccination, mice were infected with Mtb H37Rv *via* the aerosol route and IL-10 overexpression was initiated. **(A)** Lung Mtb burdens at days 15, 20, and 30 postinfection. Data represent one experiment out of three independent experiments performed, each with four to five mice per group. *p < 0.05, **p < 0.01, ^,###^p < 0.001, ****^,++++,$$$$^p < 0.0001 by one-way ANOVA followed by Tukey’s test. *, represents statistical differences between unvaccinated and BCG-vaccinated B6 mice; #, represents statistical differences between unvaccinated B6 and pMT-10 mice; +, represents statistical differences between unvaccinated pMT-10 and BCG-vaccinated B6 mice; $, represents statistical differences between unvaccinated pMT-10 and BCG-vaccinated pMT-10 mice. **(B)** Percentage of infiltrated lung area at day 30 postinfection with Mtb and representative H&E lung sections. Data are represent one experiment out of two independent experiments performed, each with four to five mice per group. *p < 0.05, **p < 0.01, ****p < 0.0001 by one-way ANOVA followed by Tukey’s test.

We began by analyzing bacterial burdens in the lung at days 15, 20, and 30 postinfection (**Figure 1A**). As expected, BCG-vaccinated B6 mice began controlling Mtb growth at day 15 postinfection (16, 28) and, by day 30 postinfection, they had 1.4 Log_10_ less bacterial burden than unvaccinated mice (**Figure 1A**). In line with our previous study (14), pMT-10 mice exhibited exacerbated lung bacterial burdens when compared with B6 mice (**Figure 1A**). Interestingly, however, BCG-vaccinated pMT-10 mice displayed significantly lower lung bacterial burdens than control pMT-10 mice. Indeed, bacterial burdens in the lungs of vaccinated pMT-10 mice were similar to control B6 mice throughout the infection period analyzed (**Figure 1A**). In accordance with these data, we found that both B6- and pMT-10-vaccinated mice had fewer areas of lesioned lung tissue than the respective control animals (**Figure 1B**). Together, these data show that BCG vaccination prevents the acute susceptibility to Mtb infection induced by IL-10 overexpression, allowing for improved control of bacterial proliferation and reduced pathology.

### IL-10 Overexpression Does Not Impair the BCG-Induced Ag85b-Specific CD4^+^ T-Cell Response Following Mtb Challenge

Mtb burdens are similar in both BCG-vaccinated and unvaccinated mice before day 15 post-challenge (28). After this time-point, control of Mtb in BCG-vaccinated mice has been shown to depend on lung-resident CD4^+^ T cells induced by BCG vaccination (16, 29). Therefore, to determine if the increased protection of vaccinated mice overexpressing IL-10 correlated with an accelerated CD4^+^ T-cell response, we first analyzed the accumulation of CD4^+^ T cells expressing the activation marker CD44 in the lungs at days 15 and 30 postinfection.

**Figure 2:**
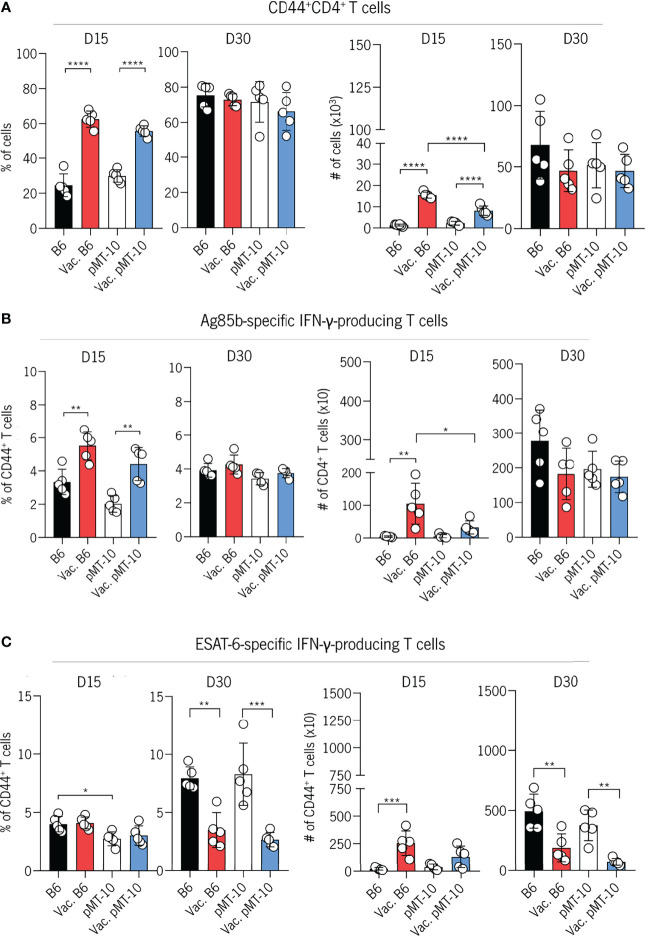
IL-10 overexpression does not impair the early accumulation of BCG-induced CD4^+^ T cells in the lungs Mtb-infected mice. B6 and pMT-10 mice were subcutaneously vaccinated with 1 × 10^6^ BCG. Unvaccinated mice were used as controls. Two months after vaccination, mice were infected with Mtb H37Rv *via* the aerosol route and IL-10 overexpression was initiated. **(A)** Frequency and number of CD4^+^ T cells expressing CD44 in the lungs of mice at days 15 and 30 postinfection. **(B)** Frequency and number of IFN-γ–producing CD4^+^ T cells after *in vitro* restimulation with the Ag85b_240-254_ peptide in the lungs of mice at days 15 and 30 postinfection. **(C)** Frequency and number of IFN-γ–producing CD4^+^ T cells after *in vitro* restimulation with the ESAT-6_1–20_ peptide in the lungs of mice at days 15 and 30 postinfection. Data represent one experiment out of two independent experiments performed, each with five mice per group. *p < 0.05, **p < 0.01, ***p < 0.001, ****p < 0.0001 by one-way ANOVA followed by Tukey’s test.

We found an increased accumulation of CD4^+^CD44^+^ T cells in the lungs of vaccinated mice at day 15 postinfection as compared to unvaccinated mice (**Figure 2A**). However, by day 30 postinfection, the frequency and number of CD4^+^CD44^+^ T cells were similar across all groups of mice (**Figure 2A**). To determine further the impact of BCG vaccination in the development of the Ag-specific response in the IL-10-enriched environment, we evaluated the frequency and number of Ag85b-specific CD4^+^ T cells and their ability to produce IFN-γ. To do this, we restimulated lung cells with Ag85b and quantified IFN-γ-producing CD4^+^ T cells at 15 and 30 days after Mtb infection (**Figure 2B**). Our data show that both groups of vaccinated mice present an increased frequency and number of Ag85b-specific IFN-γ-producing CD4^+^ T cells at day 15 postinfection when compared to control mice (**Figure 2B**). Importantly, the accumulation of Ag85b-specific IFN-γ-producing CD4^+^ T cells in the lungs of vaccinated pMT-10 mice appears to occur in a similar proportion as in vaccinated B6 mice, suggesting a minor role for IL-10 in the development of this response after BCG vaccination (**Figure 2B**). By day 30 postinfection, the frequency and number of CD4^+^ T cells producing IFN-γ in response to Ag85b were similar across all groups of mice (**Figure 2B**). These results support previous evidence that BCG-vaccinated mice exhibit an accelerated Ag85b-specific CD4^+^ T-cell response when compared to unvaccinated mice (28). Additionally, these data demonstrate that IL-10 overexpression during Mtb infection does not influence the rapid accumulation of Ag85b-specific CD4^+^ T cells in BCG-vaccinated mice.

Our previous study revealed that IL-10 delays the onset of the ESAT-6-specific CD4^+^ T-cell response. Therefore, we decided to explore the impact of IL-10 expression on the development of ESAT-6-specific CD4^+^ T-cell response in BCG-vaccinated mice. To do this, we analyzed the frequency and number of IFN-γ-producing CD4^+^ T cells in response to ESAT-6 restimulation at days 15 and 30 postinfection. We found a similar frequency and number of ESAT-6-specific IFN-γ-producing CD4^+^ T cells in the lungs of all experimental groups by day 15 postinfection. These data suggest that the increased expansion of Ag85b-specific CD4+ T cells at day 15 (**Figure 2B**) are lung-resident memory cells induced by BCG vaccination. However, at day 30 after infection, the frequency of ESAT-6-specific IFN-γ-producing CD4^+^ T cells in the lungs of both unvaccinated B6 and pMT-10 mice were higher than in vaccinated B6 and pMT-10 mice (**Figure 2C**). This decreased expansion is likely a consequence of the lower lung bacterial burdens and the stronger Ag85b-specific CD4^+^ T-cell response observed in these mice at day 15 postinfection (**Figure 2B**).

Altogether, these data demonstrate that the onset of the BCG-specific CD4^+^ T-cell response is similar in both B6- and pMT-10-vaccinated mice, thus supporting the hypothesis that IL-10 overexpression during infection does not significantly impair BCG-mediated immunity.

### BCG Vaccination Does Not Prevent the IL-10-Dependent Accumulation of CD4^+^ T Cells in the Lung Vasculature

**Figure 3:**
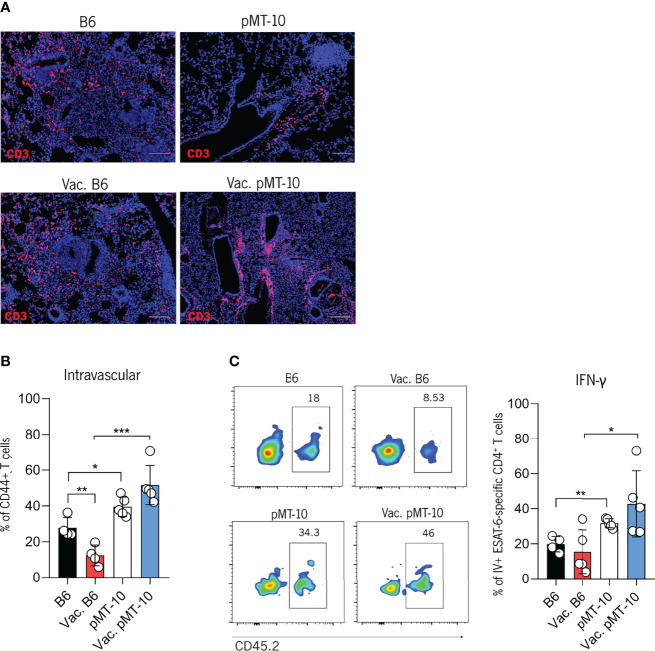
IL-10 overexpression impairs the migration of Mtb-specific CD4^+^ T cells to the lung parenchyma of Mtb-infected mice. B6 and pMT-10 mice were subcutaneously vaccinated with 1 × 10^6^ BCG. Unvaccinated mice were used as controls. Two months after vaccination, mice were infected with Mtb H37Rv *via* the aerosol route and IL-10 overexpression was initiated. **(A)** Representative immunofluorescence of CD3^+^ cells in lungs of mice at day 30 post-Mtb infection. **(B)** Frequency of intravascular (CD45^+^) CD4^+^ T cells in the lungs of mice at day 30 post-Mtb infection. **(C)** Flow cytometry analysis and frequency of intravascular (CD45^+^) ESAT-6-specific IFN-γ–producing CD4^+^ T cells in the lungs of mice at day 30 post-Mtb infection. Scale bar: 100 µm. Data represent one experiment out of two independent experiments performed, each with four to five mice per group. *p < 0.05, **p < 0.01, ***p < 0.001 by one-way ANOVA followed by Tukey’s test.

Our previous study revealed that IL-10 antagonized the control of Mtb infection by promoting the generation of vasculature-associated CD4^+^ T cells with reduced ability to migrate into the lung parenchyma and induce control of infection (14). In the present study, we showed that BCG vaccination prevents the antagonistic effects of IL-10 likely because of memory lymphocytes that are retained in the lung and rapidly respond following Mtb challenge (29). However, as BCG expresses Ag85b but not ESAT-6 we wanted to determine if BCG vaccination would overcome the impaired migration of ESAT-6-specific CD4^+^ T cells to the lung parenchyma observed in unvaccinated pMT-10 mice overexpressing IL-10 (14). Our histological analysis showed that T cells from B6 mice infiltrated the granuloma whereas in pMT-10 mice it accumulated in perivascular cuffs (**Figure 3A**). To confirm this observation and determine the location of CD4^+^ T cells in the lungs of B6 and pMT-10 mice, we performed intravital flow cytometry at day 30 postinfection. To do this, we intravenously injected mice with a fluorochrome-labeled anti-CD45.2 antibody and sacrificed them 3 min later. Lungs were then collected and processed for flow cytometry analysis, as previously described (14). Our results revealed that vaccinated B6 mice had a reduced frequency of intravascular CD4^+^CD44^+^ T cells comparatively to control B6 mice (**Figure 3B**). This finding supports previous studies, showing that the presence of parenchymal CD4^+^ T cells in the lungs of infected mice correlates with control of infection (26, 30). We also found that the frequency of intravascular CD4^+^ T cells was similar in the lungs of pMT-10 mice irrespective of their vaccination status (**Figure 3B**). Importantly, pMT-10 mice had a higher frequency of CD4^+^CD44^+^ T cells than vaccinated B6 mice, and even unvaccinated B6 mice (**Figure 3B**). The same data were found for intravascular ESAT-6-specific IFN-γ-producing CD4^+^ T cells (**Figure 3C**). Together, these data show that BCG vaccination does not overcome the IL-10-mediated migration impairment of newly activated CD4^+^ T cells to the lung parenchyma.

**Figure 4:**
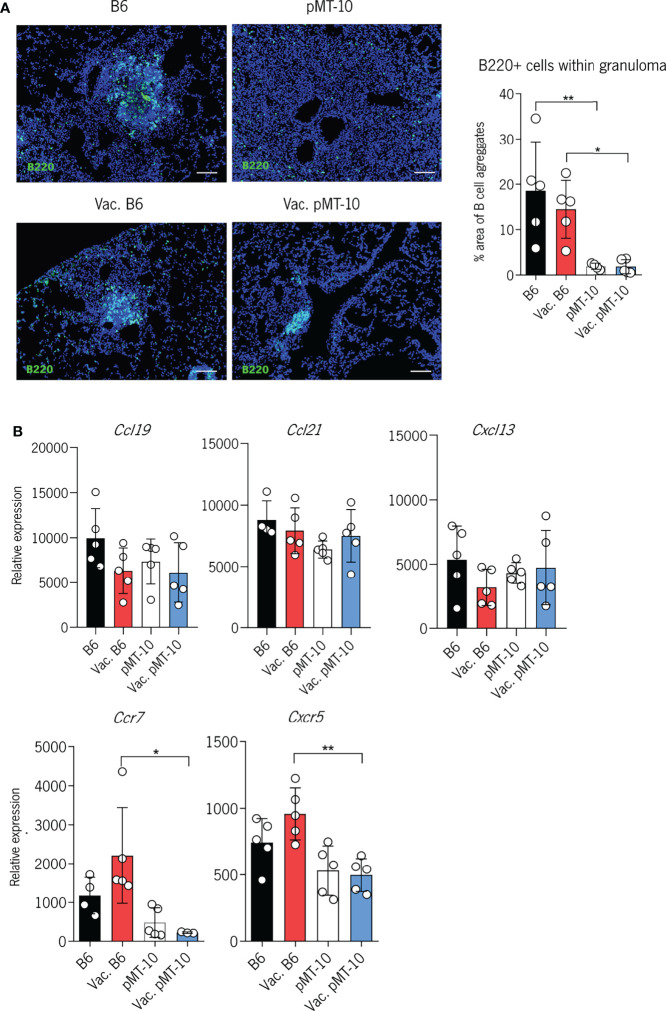
IL-10 overexpression impairs the development of B-cell follicles in the lungs of Mtb-infected mice. B6 and pMT-10 mice were vaccinated with 1 × 10^6^ BCG, and control mice were left unvaccinated. Two months after vaccination, mice were infected with Mtb H37Rv *via* the aerosol route and IL-10 overexpression was initiated. **(A)** Representative immunofluorescence of B220^+^ cells in the lungs of mice and frequency of granuloma area with B-cell aggregates at day 40 after Mtb infection. **(B)** Relative expression of homeostatic chemokines and chemokine receptors with critical roles in the formation of B-cell follicles following Mtb infection. Scale bar: 100 µm. Data represent one experiment out of two independent experiments performed, each with four to five mice per group. *P < 0.05, **P < 0.01 by one-way ANOVA followed by Tukey’s test.

It has been previously shown that the formation of perivascular cuffs in Mtb-infected lungs associates with defective formation of tertiary lymphoid follicles, as determined by the accumulation of B cells within the granuloma (17). Since our data revealed that BCG vaccination did not improve the accumulation of newly activated CD4^+^ T cells in the lung parenchyma of pMT-10 mice, we investigated the impact of BCG vaccination in the formation of tertiary lymphoid follicles. Our histological examination of lung sections of Mtb-infected lungs 30 days after infection revealed a decreased accumulation of B cells (marked with B220) in the granuloma of vaccinated and unvaccinated pMT-10 mice as compared to their B6 counterparts (**Figure 4A**). Specifically, vaccinated pMT-10 mice show smaller B-cell areas within granulomas than vaccinated B6 mice, suggesting that BCG vaccination does not overcome the IL-10-dependent impaired development of lymphoid follicles during Mtb infection.

To explore whether the impaired development of these structures was associated with reduced expression of homeostatic cytokines, we assessed the expression of CCL19, CCL21, and CXCL13 in the lungs of vaccinated and unvaccinated B6 and pMT-10 mice (**Figure 4B**). We found no significant differences in the expression of these chemokines across all groups of mice. Interestingly, however, we found that vaccinated pMT-10 mice expressed reduced levels of CCR7 and CXCR5 mRNA in the lungs when compared to vaccinated B6 mice (**Figure 4B**). These findings suggest that IL-10 expression during Mtb infection does not impair the expression of homeostatic chemokines, but it reduces the expression of chemokine receptors essential for the development of tertiary lymphoid follicles in the lungs of vaccinated mice.

In all, our data show that the presence of lung-resident Ag85b-specific T cells prior to Mtb infection is sufficient to overcome the antagonistic effects of IL-10 in the control of Mtb infection. Despite this, BCG vaccination does not appear to overcome the migration deficiency of CD4^+^ T cells primed in an IL-10-enriched environment, as is the case of ESAT-6-specific T cells, which may have a detrimental impact in the long-lasting control of infection.

## Discussion

We have recently demonstrated that IL-10 antagonizes the early control of Mtb infection by promoting the development of vasculature-associated CD4^+^ T cells with impaired ability to migrate to the lung parenchyma (14). This migratory deficit is associated with reduced antigen sensing and ability of CD4^+^ T cells to engage with infected phagocytes. On the other hand, IL-10 overexpression after the onset of the acquired response does not alter the outcome of chronic Mtb infection (14). These data show that, in the IL-10-enriched environment, CD4^+^ T cells differentiate into a vasculature-associated phenotype with reduced ability to migrate into the lung parenchyma and infiltrate the infected lesion, thereby compromising control of Mtb infection. In this work, we wanted to determine the impact of IL-10 in the control of Mtb infection in vaccinated hosts. Our data show that IL-10 overexpression during Mtb infection in BCG-vaccinated mice does not interfere with vaccine-induced protection. In both B6 (normal levels of IL-10) and pMT-10 (high levels of IL-10) BCG-vaccinated mice, the control of infection was associated with a rapid accumulation of Ag85b-specific CD4^+^ T cells in the lungs. On the other hand, there was an impaired migration of ESAT-6-specific CD4^+^ T cells to the lung parenchyma of mice overexpressing IL-10 independently of the vaccination status (14). As both Mtb and BCG express Ag85b while only Mtb expresses ESAT-6, these data show that IL-10 overexpression does not impair the recall response, likely because Ag85b-specific CD4^+^ T cells already colonize the lungs (16, 29), but it does impair the primary response. In addition, we found an impaired formation of B-cell follicles in the lungs of mice overexpressing IL-10 associated with reduced expression of CXCR5 and CCR7. Taken together, these data suggest that while an IL-10-enriched environment can modulate *de novo* differentiation and migration of recently activated CD4^+^ T cells, it does not affect already differentiated T cells.

It has been previously shown that IL-10 regulates BCG-mediated memory development and subsequent control of Mtb infection (31). Herein, we addressed the impact of IL-10 on the protective functions of an already established memory response. Our data showing a reduced impact of IL-10 in the established memory response are in line with our previously published data demonstrating that IL-10 overexpression during the chronic stage of Mtb infection does not impact the outcome of infection (14). These observations together suggest that the impact of IL-10 in disease progression is minimal after the onset of the CD4^+^ T-cell response. Indeed, our previous data showed that the antagonistic role of IL-10 in the control of Mtb infection was mainly in the development of vasculature-associated CD4^+^ T cells that lack the ability to migrate to the lung parenchyma and engage with infected phagocytes (14). Therefore, these observations taken together with previous data showing that protection conferred by BCG vaccination is dependent on memory lymphocytes retained in the lung (29) suggest that the reduced impact of IL-10 in vaccinated mice likely occurs because a subset of memory lymphocytes is already colonizing the lungs. In accordance with these data, we found a similar expansion of Ag85b-specific T-cell responses at day 15 post-Mtb infection in both B6- and pMT-10-vaccinated hosts. It is also a possibility that memory T cells generated by BCG vaccination are more resilient to the immunosuppressive effects of IL-10. However, it has been recently demonstrated that mice vaccinated with the second-generation recombinant BCG30 vaccine and treated with the molecule inhibitor of the IL-10/STAT3 axis, 5,15-diphenyl porphyrin (DPP), after challenge with Mtb aerosol infection resulted in the increased proliferation of central and effector memory T cells compared with untreated mice (32). These findings suggest that IL-10 signaling is similar in both naïve and memory T cells. As such, our results are best explained by the protective effect of lung-resident CD4^+^ T cells that rapidly control Mtb infection. Additionally, they further support our previous data showing that the main antagonistic effect of IL-10 is in the development of vasculature-associated CD4^+^ T cells (14).

A direct interaction between CD4^+^ T cells and infected phagocytes is essential to controlling Mtb growth (15). Recent evidence suggests that the migration of T cells into the lung parenchyma, specifically CXCR5-expressing T cells, is associated with the development of ectopic lymphoid structures characterized by the formation of B-cell follicles. The formation of these ectopic structures has been suggested to facilitate optimal positioning of T cells promoting the development of a more protective granuloma (17). Indeed, a defective development of ectopic lymphoid structures is associated with poor control of infection both in humans and in experimental animal models of Mtb infection (17, 21, 33). Despite this, the precise mechanisms whereby ectopic lymphoid structures promote better control of infection remain unclear. Our study supports the association between reduced T-cell infiltration in the lung parenchyma and the defective formation of ectopic lymphoid structures, suggesting that recently activated CD4^+^ T cells, rather than memory T cells, play a key role in the development of these structures. However, it has been recently demonstrated that group 3 innate lymphoid cells expressing CXCR5 also play a crucial role in the formation of ectopic lymphoid structures and in the control of Mtb infection (34). IL-10 may also modulate the activity of these innate lymphocytes to hamper the generation of B-cell follicles. While we did not analyze the activity nor the accumulation of group 3 innate lymphoid cells, we did find reduced expression of CXCR5 and CXCR7 in the lungs of pMT-10 mice. Nevertheless, the fact that BCG vaccination confers early protection in these mice shows that ectopic lymphoid structures are not essential for the early control of infection. However, it is possible that the long-term control of infection is not maintained in the absence of ectopic lymphoid follicles. Future experiments are required to address this question.

BCG vaccination has been shown to confer 60–80% protective efficacy against tuberculous meningitis in children (4); however, its efficacy against adult pulmonary TB is highly variable (35). While this work aimed to mechanistically define the impact of IL-10 overexpression in the T-cell response specifically after vaccination, these data may have important clinical implications, specifically in individuals exposed to Mtb that may have coinfection with SARS-CoV-2 (36), influenza (37), or malaria (38) and consequently produce high IL-10. Our data suggest that IL-10 overexpression in these patients should not impair BCG-induced protection; however, if indeed the development of B-cell follicles is critical for long-term control of Mtb infection, the overexpression of IL-10 may contribute to the reactivation of Mtb infection. Future research is necessary to clarify this issue.

In addition to IL-10, other immunoregulatory molecules and pathways restrict the migration and protective function of CD4^+^ T cells during Mtb infection. For example, TGFβ impairs the production of IFN-γ by CD4^+^ T cells within the granuloma (39). Indoleamine 2,3-dioxygenase (IDO1) prevents the migration of CD4^+^ T cells to the granuloma and impairs the development of lymphoid follicles (40). These data, together with our data, underlie the importance of understanding further the role of immunoregulatory pathways and their impact following vaccination and during Mtb infection. This understanding will likely contribute to the development of novel and more effective vaccines and therapies against TB.

Overall, our data show that BCG vaccination prevents the antagonistic effects of IL-10 in the early control of Mtb infection. Despite this, the primary CD4^+^ T-cell response elicited by antigens not expressed by BCG remains affected by IL-10, resulting in increased CD4^+^ T-cell accumulation in the lung vasculature and formation of smaller B-cell follicles.

## Data Availability Statement

The raw data supporting the conclusions of this article will be made available by the authors, without undue reservation.

## Ethics Statement

The animal study was reviewed and approved by Subcomissão de Ética para as Ciências da Vida e da Saúde (SECVS 074/2016) and the Portuguese National Authority Direcção Geral de Veterinária (DGAV 014072).

## Author Contributions

CF, AGC, and ET conceived and designed the study. CF, CM, AB, and PB-S performed the experimental work and data analysis. MR, MV, RS, CC, AC, FR, and MC-N provided intellectual and technical and/or material support. CF, AGC, and ET drafted the manuscript. AGC and ET acquired funding. All authors critically revised and approved the manuscript and accepted accountability.

## Funding

This work was supported by national funds through the Foundation for Science and Technology (FCT) projects PTDC/SAU-INF/28463/2017, PTDC/MED-ONC/28658/2017, UIDB/50026/2020, and UIDP/50026/2020; by the Northern Portugal Regional Operational Programme (NORTE 2020), under the PORTUGAL 2020 Partnership Agreement, through the European Regional Development Fund (ERDF) HEALTH-UNORTE (NORTE-01-0145-FEDER-000039); and by the ICVS Scientific Microscopy Platform, member of the national infrastructure PPBI - Portuguese Platform of Bioimaging (PPBI-POCI-01-0145-FEDER-022122). ET, RS, and CC were supported by the FCT Estímulo Individual ao Emprego Científico 2020.03070.CEECIND, 2020.00185.CEECIND, and 2018.04058.CEECIND, respectively; CF, CM, and AB by FCT PhD fellowships PD/BD/137447/2018, 2020.05976.BD, and SFRH/BD/120371/2016, respectively.

## Conflict of Interest

The authors declare that the research was conducted in the absence of any commercial or financial relationships that could be construed as a potential conflict of interest.

## Publisher’s Note

All claims expressed in this article are solely those of the authors and do not necessarily represent those of their affiliated organizations, or those of the publisher, the editors and the reviewers. Any product that may be evaluated in this article, or claim that may be made by its manufacturer, is not guaranteed or endorsed by the publisher.
